# Protective effects of Dioscin against sepsis‐induced cardiomyopathy via regulation of toll‐like receptor 4/MyD88/p65 signal pathway

**DOI:** 10.1002/iid3.1229

**Published:** 2024-05-22

**Authors:** Meng Zhang, Deyuan Zhi, Pei Liu, Yajun Wang, Meili Duan

**Affiliations:** ^1^ Department of Critical Care Medicine, Beijing Friendship Hospital Capital Medical University Beijing China

**Keywords:** Dioscin, lipopolysaccharide, MyD88, sepsis‐induced cardiomyopathy, TLR4

## Abstract

**Background:**

Dioscin has many pharmacological effects; however, its role in sepsis‐induced cardiomyopathy (SIC) is unknown. Accordingly, we concentrate on elucidating the mechanism of Dioscin in SIC rat model.

**Methods:**

The SIC rat and H9c2 cell models were established by lipopolysaccharide (LPS) induction. The heart rate (HR), left ventricle ejection fraction (LVEF), mean arterial blood pressure (MAP), and heart weight index (HWI) of rats were evaluated. The myocardial tissue was observed by hematoxylin and eosin staining. 4‐Hydroxy‐2‐nonenal (4‐HNE) level in myocardial tissue was detected by immunohistochemistry. Superoxide dismutase (SOD), catalase (CAT), and glutathione (GSH) activities in serum samples of rats and H9c2 cells were determined by colorimetric assay. Bax, B‐cell lymphoma‐2 (Bcl‐2), toll‐like receptor 4 (TLR4), myeloid differentiation primary response 88 (MyD88), phosphorylated‐p65 (p‐p65), and p65 levels in myocardial tissues of rats and treated H9c2 cells were measured by quantitative real‐time PCR and Western blot. Viability and reactive oxygen species (ROS) accumulation of treated H9c2 cells were assayed by 3‐(4,5‐dimethylthiazol‐2‐yl)‐2,5‐diphenyltetrazolium bromide and dihydroethidium staining assays.

**Results:**

Dioscin decreased HR and HWI, increased LVEF and MAP, alleviated the myocardial tissue damage, and reduced 4‐HNE level in SIC rats. Dioscin reversed LPS‐induced reduction on SOD, CAT, GSH, and Bcl‐2 levels, and increment on Bax and TLR4 levels in rats and H9c2 cells. Overexpressed TLR4 attenuated the effects of Dioscin on promoting viability, as well as dwindling TLR4, ROS and MyD88 levels, and p‐p65/p65 value in LPS‐induced H9c2 cells.

**Conclusion:**

Protective effects of Dioscin against LPS‐induced SIC are achieved via regulation of TLR4/MyD88/p65 signal pathway.

## Graphical abstract

(1) Dioscin improved the myocardial tissue damage of SIC rat. (2) Dioscin improved LPS‐decreased the activities of SOD, CAT, and GSH. (3) Overexpressed TLR4 promoted the Dioscin‐suppressed the viability of H9c2 cell.

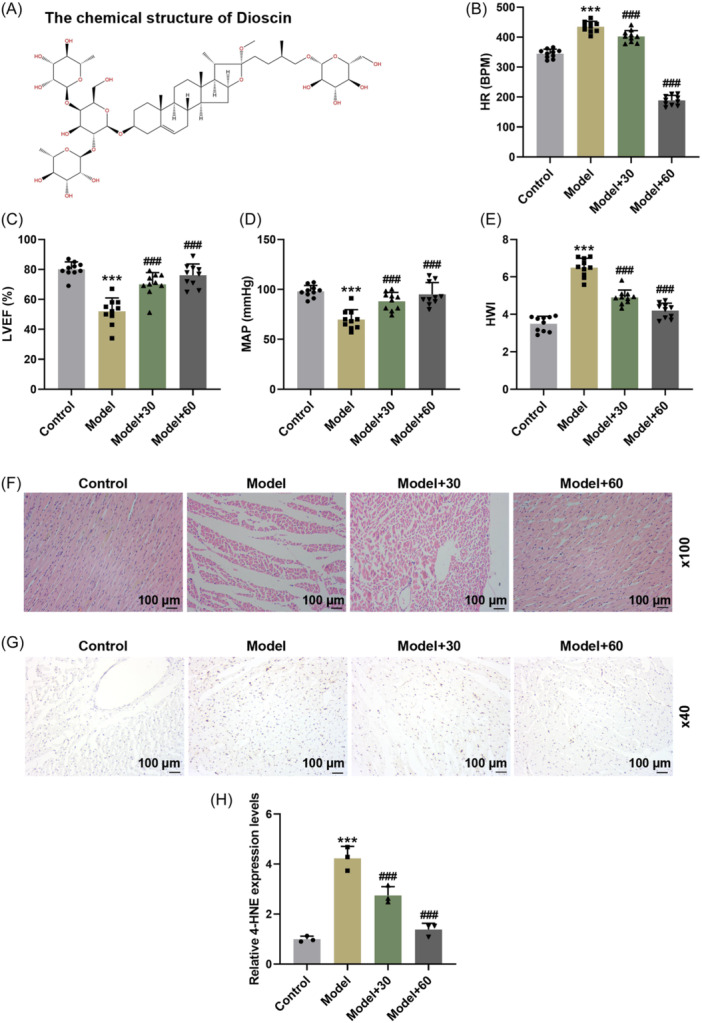

## 1 INTRODUCTION

Sepsis refers to a common complication caused by various injuries, extracorporeal membrane oxygenation, and chronic hemodialysis access, further development of which can lead to septic shock, one of the important causes of death in critically ill patients.^1^, ^2^, ^3^, ^4^ Studies have shown that sepsis is essentially an inflammation‐mediated host autoimmune injury characterized by the systemic inflammatory response syndrome.^5^, ^6^ Sepsis‐induced cardiomyopathy (SIC) may be consequent on severe sepsis, and myocardial injury is also one of the common complications of sepsis.^7^, ^8^, ^9^ According to relevant reports, SIC can cause severe heart failure, heart dysfunction and endotoxemic shock, and aggravate hypoxic‐ischemic damage of other tissues and organs throughout the body.^10^, ^11^, ^12^ At present, the main molecular mechanism of SIC has been confirmed as mitochondrial dysfunction causing oxidative stress, which later triggers cellular damage.^13^ In addition, since SIC frequently results in heart dysfunction, the detection of hemodynamic parameters can aid in assessing the pump function and myocardial contractility of the heart.^14^

Sepsis, also known as bloodstream infection, features the presence of harmful microorganisms, such as bacteria or fungi.^15^ This systemic inflammation stimulates immune cells within the heart, leading to the release of excessive proinflammatory cytokines, including tumor necrosis factor‐α (TNF‐α) and interleukin‐1β (IL‐1β).^16^ Notably, these cytokines can directly damage cardiomyocytes and interfere with their normal contractile function, thereby exacerbating cardiac dysfunction.^17^ The immune‐inflammatory response in SIC can also bring about oxidative stress.^18^ It was known that oxidative stress can damage cardiomyocytes and negatively affect their abilities to function properly.^19^ Control of the immune‐inflammatory response is critical for preventing and treating SIC. Therefore, our study attempts to figure out whether modulating immune‐inflammatory response‐induced oxidative stress can mitigate the progression of SIC.

Currently, timely use of antibiotics is considered the cornerstone in the treatment of sepsis.^20^ In addition, with the rapid development of pharmaceutical analysis, more and more traditional Chinese medicines are being used for the treatment of SIC, such as Xuebijing injection, Paeoniflorin, and Resveratrol.^13^ Dioscin is a natural steroidal saponin derived from *Dioscorea nipponica* Makino, which has been identified by modern pharmacological studies to exert numerous pharmacological effects, including antitumor and antiarthritic properties.^21^, ^22^ Recent reports have revealed the protective role of Dioscin in cardiovascular and cerebrovascular systems.^23^, ^24^ Qin et al. have demonstrated that Dioscin can effectively inhibit ischemia/reperfusion‐induced apoptosis of rat cardiomyocytes, increase the level of superoxide dismutase (SOD), and reduce the level of intracellular reactive oxygen species (ROS).^25^ However, the role of Dioscin in SIC still awaits investigation.

Toll‐like receptor 4 (TLR4), an essential lipopolysaccharide (LPS) signaling receptor, is instrumental in the activation of innate immunity.^26^ TLR4 impacts the immune system by recognizing and binding molecules, like LPS, from bacteria, to activate downstream signaling pathways, such as the nuclear factor‐kappa B (NF‐κB) and mitogen‐activated protein kinase (MAPK) pathways, which subsequently induces the expressions of inflammation‐related genes and promotes the production of cytokines, including TNF‐α and IL‐1β.^27^, ^28^ However, the overactivated TLR4 pathway may lead to the development of chronic inflammation‐related diseases, comprising asthma, rheumatoid arthritis, inflammatory bowel disease and SIC.^8^, ^22^, ^29^, ^30^ Chen et al. found that losartan regulates macrophage polarization via TLR4‐mediated NF‐κB and MAPK signaling pathways, thereby reducing oxidative stress and cardiomyocyte apoptosis, and ultimately attenuating SIC.^31^ Besides, TLR4 can interact with the adaptor protein myeloid differentiation primary response 88 (MyD88) to activate NF‐κB p65, which is indispensable for the production of cytokines such as IL‐6, IL‐8, and TNF‐α.^32^, ^33^ MyD88 is an intracellular adapter protein that coordinates proinflammatory signaling cascades.^34^ Notably, Dioscin ameliorates acute lung injury (ALI) through inhibiting the TLR4/MyD88 signaling pathway,^35^ in which inflammation and oxidative stress are involved.^36^ Therefore, we speculated that Dioscin may also play a protective role against SIC by modulating TLR4/MyD88 signaling pathway.

In this study, we established the SIC rat model by intra‐venous injection of LPS to investigate the effect of Dioscin and its mechanism of action in the SIC. This study innovatively probed into the role of Dioscin in SIC through the TLR4/MyD88/p65 signaling pathway, which not only deepens our comprehension of SIC progression, but also provides new research directions for future interventions of cardiac dysfunction.

## 2 MATERIALS AND METHODS

### 2.1 Animal models and grouping

Forty male Sprague‐Dawley rats (10‐week‐old, 300 ± 25 g) were ordered from the ALF Biotechnology Co., Ltd. (Jiangsu, China) 1 week before the study commenced. Rats were kept in the experimental animal room at room temperature with a 12 h light/dark cycle under 50%–60% humidity, and had free access to water and feed. In this study, all rats were randomly divided into the following four groups (*n* = 10/group): (1) Control group, (2) Model group, (3) Model + 30 group, (4) Model + 60 group.

As previously reported,^37^ SIC model rats were established by intravenous injection of 5 mg/kg LPS (GC38505; GlpBio), and rats in the Control group were injected with the same volume of saline (PB180353; Procell). One hour later, SIC model rats received 30/60 mg/kg Dioscin (D114066; Aladdin) through intragastric administration in the Model + 30 group and Model + 60 group, while rats in the Control and Model groups were treated with the same volume of saline. Three hours after LPS or saline injection, all rats were anesthetized by 2% volume of isoflurane (1349003; Merck) and 1 L/min O_2_, followed by examination of in vivo hemodynamic parameters. Next, all rats were euthanized using intraperitoneal injection of pentobarbital sodium (150 mg/kg; P‐010; Supelco), and killed by cervical dislocation. Then, rat heart tissues, myocardial tissues, and serum were collected for heart and body weight (BW) detection, hematoxylin and eosin (H&E) staining, immunohistochemistry (IHC) analysis, and Western blot.

### 2.2 Detection of hemodynamic parameters

As previously described,^37^ the heart rate (HR), left ventricle ejection fraction (LVEF), and mean arterial blood pressure (MAP) of all rats were measured utilizing the Vevo 2100 imaging system (VisualSonics) equipped with a 10 MHz sectorial probe.

Following anesthesia, the rats were immobilized on the experimental table. The chest of the rats was shaved and the sectorial probe was positioned to acquire images of cardiac contraction and relaxation. A clear cardiac cycle displaying both systole and diastole was selected on the software. The HR, LVEF, and MAP were automatically calculated using the software VisualSonics Vevo LAB (VisualSonics).

### 2.3 Determination of heart weight index

According to an existing report,^38^ the heart weight index (HWI) of rats was tested. After anesthesia, the BW of all rats was measured using the electronic analytical balance (E0261; Beyotime), and then rats were killed by cervical dislocation. Next, the heart tissues of rats were collected (excluding connective tissues and large blood vessels) and blotted with filter paper (FFT08; Beyotime), after which the heart weight (HW) was measured using the electronic analytical balance. The HWI was computed as HWI = HW/BW.

### 2.4 H&E staining

One hundred percent ethanol (E130059) and xylene (X139941) were ordered from Aladdin, and the gradient ethanol (70%, 85%, 95%, and 100%) was prepared using 100% ethanol. The rat myocardial tissues were collected, fixed in 4% paraformaldehyde (P0099; Beyotime) for 24 h at room temperature, dehydrated using gradient ethanol (70%, 85%, 95%, and 100%) for 3 min, transparentized with xylene, embedded into paraffin, and sliced into 5‐μm thick sections. After that, the sections were immersed in xylene solution for 30 min, hydrated by different concentrations of ethanol (100%, 95%, 85%, and 70%) for 3 min, and washed for 5 min with water. Subsequently, the sections were stained with hematoxylin staining solution (C0107; Beyotime) for 10 min, and eosin staining solution (C0109; Beyotime) for 5 min, followed by 3‐min dehydration with gradient ethanol (70%, 85%, 95%, and 100%), and 3‐min treatment with xylene. Finally, the sections were mounted using neutral gum (N116470; Aladdin) and photographed using an inverted microscope (×100 magnification; Ts2R‐FL; Nikon).

### 2.5 IHC analysis

IHC Kit (ENS004) was purchased from NeoBioScience. The tissue sections were prepared as above, immersed in 3% H_2_O_2_‐methanol mix solution for 10 min, and treated with blocking buffer and goat serum mix solution for 10 min at room temperature. Next, the tissue sections were cultured with diluted 4‐hydroxy‐2‐nonenal (4‐HNE) mouse primary antibody (25 μg/mL; MAB6115; Abnova) overnight at 4°C, and with goat anti‐mouse secondary antibody (1:1000; ab6789; Abcam) for 30 min at 37°C. Then, the tissue sections were treated with DAB chromogenic solution, and counterstained with hematoxylin staining solution, followed by differentiation with hydrochloric acid alcohol. Finally, tissue sections were dehydrated, cleared, mounted, and photographed using an inverted microscope under ×40 magnification.

### 2.6 Cell culture

Rat‐derived H9c2 cardiomyocytes (CL‐0089) and the special medium for H9c2 cells (CM‐0089) were ordered from Procell. H9c2 cells were maintained in the special culture medium at 37°C with 5% CO_2_ in the incubator (HH.CP‐T, Grows Instrument Co., Ltd), with the culture medium changed every 2 days.

### 2.7 Cell transfection and treatment

The rat H9c2 cells were collected and the cell concentration was adjusted. Then, cells were seeded in a six‐well plate (2.5 × 10^5^ cells/well). The Lipofectamine 2000 Reagent Kit (11668027) was obtained from the Thermo Fisher Scientific. Plasmids for overexpressing TLR4 and its negative control (NC, pcDNA3.1 vector) were synthesized from Genepharma. For cell transfection, when the cell confluence reached about 80%, H9c2 cells were transfected with overexpressing TLR4 plasmids or its NC for 48 h under the help of Lipofectamine 2000 Reagent Kit.

According to the previous experimental methods,^39^, ^40^ Dioscin was dissolved in dimethyl sulfoxide (DMSO; ST1276; Beyotime) and the vehicle concentration of DMSO was <0.1%. H9c2 cells after transfection or not were collected and maintained in culture medium containing 10 μg/mL of LPS for 24 h, together with/without 200 ng/mL of Dioscin for 24 h.

### 2.8 Cell viability assay

Treated H9c2 cells were inoculated in 96‐well plates (2000 cells/well) and cultured in an incubator (37°C, 5% CO_2_) for 24 h. The 3‐(4,5‐dimethylthiazol‐2‐yl)‐2,5‐diphenyltetrazolium bromide (MTT) cell proliferation assay kit (G020‐1‐1; Nanjing Jiancheng Bioengineering Institute) was used to assess the viability of transfected H9c2 cells. Briefly, treated cells were treated with 50 μL MTT solution at 37°C for 4 h, followed by incubation with 150 μL DMSO after removal of the supernatant. Later, the optical density of each well at 570 nm was measured using a microplate reader (Bio‐Rad).

### 2.9 Colorimetric assay

SOD assay kit (A001‐3‐2), catalase (CAT) assay kit (A007‐1‐1), and glutathione (GSH) assay kit (A006‐2‐1) were ordered from Nanjing Jiancheng Bioengineering Institute. The serum samples and cell lysates were collected and reacted with corresponding reagents at 37°C (20 min for SOD detection), and the optical density of each well at 450 nm (for SOD) and 405 nm (for CAT) was measured by a microplate reader to assess the activities of SOD and CAT.

To test the level of GSH, the Reagents 1, 2, 3, and 4 were prepared using the GSH assay kit. Fifty microliters serum samples and 100 μL cell lysates were treated with Reagent 1, followed by centrifugation. Then, 100 μL supernatant was collected, and cultured with Reagent 2 (100 μL) and Reagent 3 (25 μL) for 5 min at room temperature. The absorbance of each well at 405 nm was measured by a microplate reader.

### 2.10 Detection of ROS level

The level of ROS was measured using the dihydroethidium (DHE)‐ROS detection kit (HR8685; Baiaolaibo Technology Co., Ltd). Concretely, the DHE was diluted with fresh culture medium at a ratio of 1:500. After removal of the culture medium, the cells were washed with phosphate‐buffered saline (C0221A; Beyotime), and then incubated with the appropriate volume of diluted DHE in the dark at 37°C for 60 min. Ultimately, cells were washed with fresh culture medium and observed under a fluorescence microscope (MVX10; OLYMPUS) at ×200 magnification.

### 2.11 RNA isolation and quantitative real‐time polymerase chain reaction

QRT‐PCR was performed with reference to a previous study.^41^ Total RNA was extracted from H9c2 cells by the TransZol Up Plus RNA Kit (ER501‐01; TransGen Biotech).^42^ Next, the isolated RNA sample concentration was evaluated using a spectrophotometer (Cary 60 UV‐Vis; Agilent), and cDNA was synthesized using RNA as template with the help of a One‐Step RT‐PCR SuperMix kit (AE411‐02; TransGen Biotech). After that, qRT‐PCR reaction solution was prepared by the Top Green qPCR SuperMix kit (AQ131‐01; TransGen Biotech) and supplemented with above cDNA and corresponding primers. Then, the PCR reaction was performed on the qRT‐PCR system (ABI7700; Applied Biosystems) under the following conditions: 1 cycle at 94°C for 30 s, 40 cycles at 94°C for 5 s, 40 cycles at 60°C for 15 s, and 40 cycles at 72°C for 10 s. The results were analyzed by the $2^{-∆∆C_{t}}$ method,^43^ and primer sequences of TLR4 and glyceraldehyde 3‐phosphate dehydrogenase (GAPDH, the endogenous control) were listed in Table 1.

**Table 1:** All primers in qRT‐PCR experiments in this study.

ID	Forward sequence (5′–3′)	Reverse sequence (5′–3′)
TLR4	CGCTTTCAGCTTTGCCTTCATTAC	AGCTACTTCCTTGTGCCCTGTGAG
GAPDH	CAATGACCC CTTCATTGACC	TTGATTTTGGAGGGATCTCG

### 2.12 Western blot

According to a previous description, the levels of Bax, B‐cell lymphoma‐2 (Bcl‐2), TLR4, MyD88, p‐p65, and p65 were detected.^44^ Total protein was extracted from H9c2 cells and myocardial tissues with the help of Total protein extraction kit (BB‐3101; BestBio), the concentration of which was quantified using the Protein quantitative kit (BCA; DQ111‐01; TransGen Biotech). Afterwards, sodium dodecyl sulfate–polyacrylamide gel (SDS‐PAGE) was prepared by SDS‐PAGE gel preparation kit (BB‐3702; BestBio), and 20 μL of protein samples were electrophoresed. The separated proteins were transferred onto the polyvinylidene fluoride membrane (LC2002; Thermo Fisher Scientific) with Western Transfer Buffer (BB‐35112; BestBio). Subsequently, the membrane was blocked with Western Blocking Buffer (BB‐3512; BestBio) at room temperature for 1 h, and incubated with the primary antibody working dilution at 4°C overnight. Post washing with Western Wash Buffer (P0023C3; Beyotime), the membrane was incubated with secondary antibodies at room temperature for 1 h, rinsed with Western Wash Buffer, and visualized by ECL working solution (32209; Thermo Fisher Scientific). Finally, the Western blot imaging system (FluorChem M; Alpha Innotech) and ImageJ software (Version. 5.0; Bio‐Rad) were used to analyze the results of Western blot. In this test, all information of antibodies was listed in Table 2.

**Table 2:** All antibodies information and sources in Western blot in this study.

ID	Catalog number	Company (country)	Molecular weight	Dilution ratio
Bax	ab32503	Abcam (Cambridge, UK)	21 kDa	1/1000
Bcl‐2	ab194583	Abcam (Cambridge, UK)	26 kDa	1/1000
TLR4	A00017S441	Boster Bio‐Engineering (Santa Cruz, CA, USA)	95 kDa	1/1000
MyD88	ab219413	Abcam (Cambridge, UK)	33 kDa	1/1000
p‐p65	ab76302	Abcam (Cambridge, UK)	65 kDa	1/1000
p65	ab16502	Abcam (Cambridge, UK)	64 kDa	1/1000
GAPDH	ab181602	Abcam (Cambridge, UK)	36 kDa	1/10000
Rabbit IgG	ab205718	Abcam (Cambridge, UK)		1/5000

### 2.13 Statistical analysis

In this study, GraphPad Prism8.0 (GraphPad Software) was used for statistical analysis. Measurement data were expressed as mean ± standard deviation. Statistical significance of differences was assessed with repeated measures analysis of variance (ANOVA) for multiple measurements of the same group at different time points or conditions. Comparisons among multiple groups were performed using one‐way ANOVA, followed by Tukey post hoc test. *p* < .05 was considered statistically significant.

## 3 RESULTS

### 3.1 Dioscin decreased HR and HWI, and increased LVEF and MAP in SIC rat model

**Figure 1:**
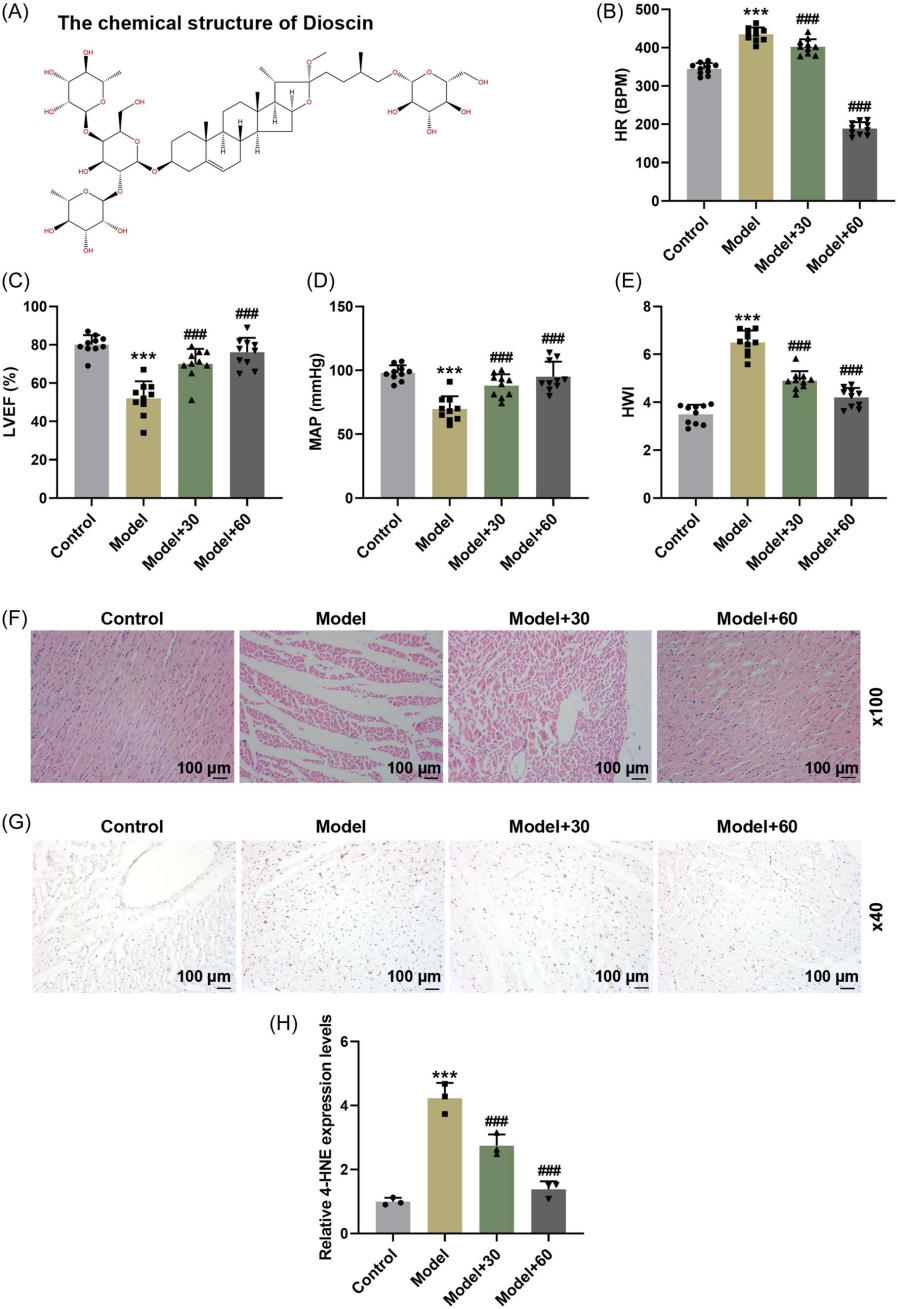
Dioscin decreased heart rate (HR) and heart weight index (HWI), increased left ventricle ejection fraction (LVEF) and mean arterial blood pressure (MAP), improved the myocardial tissue damage, and reduced 4‐hydroxy‐2‐nonenal (4‐HNE) level in myocardial tissues of sepsis‐induced cardiomyopathy (SIC) rat model. (A) The chemical structure of Dioscin. (B–E) The SIC rat model was established by intravenous injection of LPS. HR (B), LVEF (C), and MAP (D) of rats were evaluated using the Vevo 2100 imaging system, and then the HWI (E) was detected using the electronic analytical balance. (F–H) Rat myocardial tissues were observed by hematoxylin and eosin staining (F) (under ×100 magnification, scale bar = 100 μm), and the 4‐HNE level in myocardial tissue was detected by immunohistochemistry (G) (under ×40 magnification, scale bar = 100 μm); (H) the quantitative analysis of the 4‐HNE level in myocardial tissue (***p < .001 vs. control; ^###^ *p* < .001 vs. model; *n* = 10, one‐way analysis of variance was utilized to compare difference among multiple groups, followed by Tukey post hoc test).

In this study, we established the SIC rat model to investigate the role of Dioscin (chemical structure in Figure 1A). By examining hemodynamic parameters in SIC rat model, we found that the HR was obviously increased by intravenous injection of LPS, but was later decreased with the addition of Dioscin (Figure 1B, *p* < 0.001).

In the meantime, the LVEF and MAP in SIC rat model were confirmed to be reduced, while the two indexes were elevated following the treatment of Dioscin (Figure 1C,D, *p* < .001). Besides, we evaluated the HWI in rats, and confirmed that Dioscin counteracted the effects of LPS on increasing HWI, and diminished HWI in SIC rat model (Figure 1E, *p* < .001). The above results indicated that Dioscin reversed the effects of LPS on HR, HWI, LVEF, and MAP in SIC rats.

### 3.2 Dioscin improved the myocardial tissue damage, and reduced 4‐HNE level in SIC rat model

We found from Figure 1F that after SIC modeling through induction of LPS, in the myocardial tissues, inflammatory cell infiltration and myocardial fiber rupture were observed; however, the myocardial tissue damage could be improved following the treatment with Dioscin. Moreover, LPS augmented 4‐HNE level in rats, which was partially neutralized by Dioscin (Figure 1G,H). These findings further demonstrated the protective effect of Dioscin on SIC rat myocardial tissue.

### 3.3 Dioscin offset the effects of LPS on decreasing SOD, CAT, and GSH activities and Bcl‐2 level, and increasing Bax and TLR4 levels in SIC rat model

**Figure 2:**
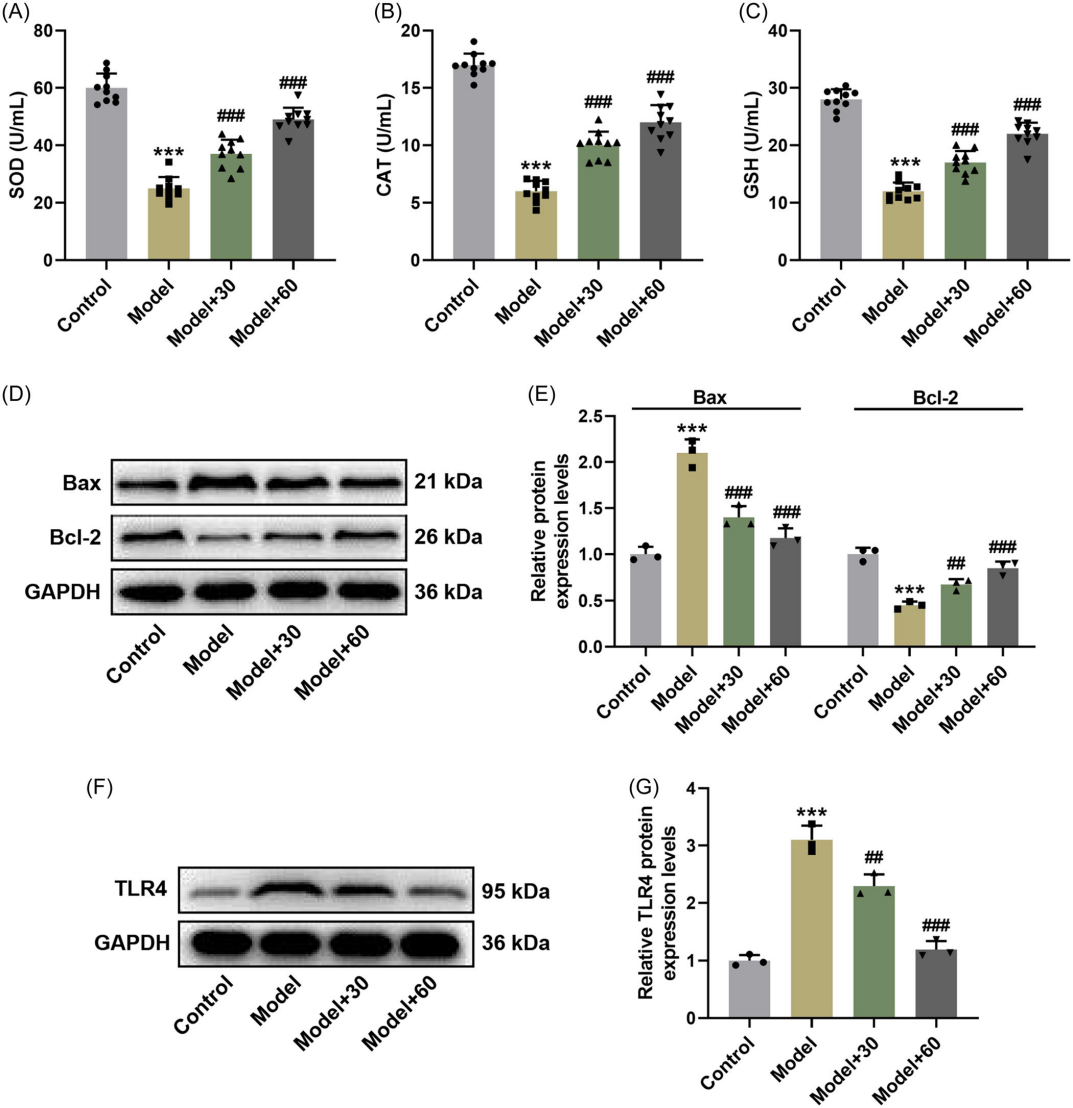
Dioscin reversed the effects of lipopolysaccharide on decreasing superoxide dismutase (SOD), catalase (CAT), and glutathione (GSH) activities and B‐cell lymphoma‐2 (Bcl‐2) level, and increasing Bax and oll‐like receptor 4 (TLR4) levels in sepsis‐induced cardiomyopathy (SIC) rat model. (A–C) The serum samples of SIC rat model were collected, and then the activities of SOD (A), CAT (B), and GSH (C) were assessed using colorimetric assay. (D–G) The levels of Bax and Bcl‐2 (D, E), and TLR4 expression (F, G) in myocardial tissues were determined by Western blot, and glyceraldehyde 3‐phosphate dehydrogenase (GAPDH) was used as an internal control (***p < .001 vs. control; ^##^ *p* < .01; ^###^ *p* < .001 vs. model; *n* = 10, one‐way analysis of variance was utilized to compare difference among multiple groups, followed by Tukey post hoc test).

To validate the above experimental results, we further explored the effect of Dioscin on the activities of SOD, CAT, and GSH in LPS‐induced SIC rat model. The results indicated that the activities of SOD, CAT, and GSH in model group were significantly lessened, as compared with those in control group (Figure 2A–C, *p* < .001); however, reduction of the three indexes in model group was reversed following treatment with Dioscin (Figure 2A–C, *p* < .001). These results implied that oxidative stress was involved in the development of SIC. Next, we also evaluated the levels of Bax and Bcl‐2 in tissues samples of SIC rat model. The data mirrored that Bax level was notably raised yet Bcl‐2 level was lessened in rat myocardial tissues after LPS induction (Figure 2D,E, *p* < .001), whereas Dioscin attenuated the role of LPS by augmenting Bcl‐2 level and diminishing Bax level (Figure 2D,E, *p* < .01), which signified that cardiomyocyte apoptosis was associated with the development of SIC. In addition, we detected the protein level of TLR4 in myocardial tissues of LPS‐induced SIC rat model. As illustrated in Figure 2F,G, LPS markedly increased TLR4 level, but Dioscin mitigated the impacts of LPS upon TLR4 level in SIC rat model (Figure 2F,G, *p* < .01). These data manifested the strong relationship between TLR4 level and myocardial tissue injury in rats.

### 3.4 Overexpressed TLR4 weakened the impacts of Dioscin upon decreasing the levels of TLR4 and ROS, and promoting viability of LPS‐induced H9c2 cells

**Figure 3:**
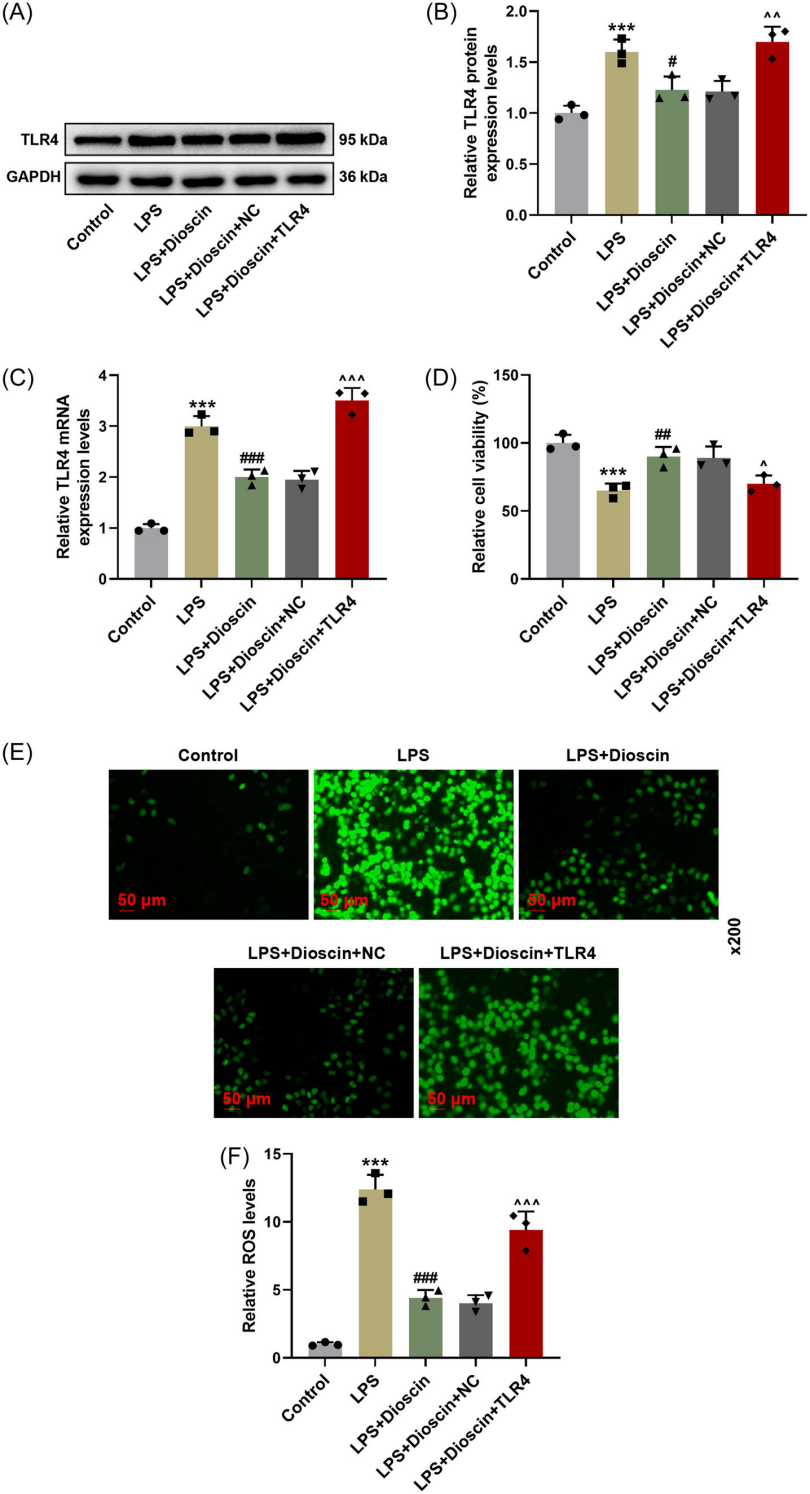
Overexpressed toll‐like receptor 4 (TLR4) offset the role of Dioscin in reducing levels of TLR4 and reactive oxygen species (ROS), and enhancing viability of lipopolysaccharide (LPS)‐induced H9c2 cells. (A–F) The H9c2 cells were transfected with overexpressing TLR4 plasmids or its negative control (NC), and the cells were treated with LPS and/or Dioscin for 24 h. TLR4 level was measured by Western blot (A, B) and quantitative real‐time polymerase chain reaction (C), and GAPDH was used as an internal control. The viability of the treated H9c2 cells was tested using MTT assay (D), and the production of ROS was evaluated in treated H9c2 cells by dihydroethidium staining assay (E‐F) (under ×200 magnification, scale bar = 50 μm) (***p < .001 vs. control; ^#^ *p* < .05; ^##^ *p* < .01; ^###^ *p* < .001 vs. LPS; ^^^ *p* < .05; ^^^^ *p* < .01; ^^^^^ *p* < .001 vs. LPS + Dioscin + NC; *n* = 3, one‐way analysis of variance was utilized to compare difference among multiple groups, followed by Tukey post hoc test, repeated measures analysis of variance was utilized for multiple measurements of the same group at different conditions).

Similarly, we also found that LPS obviously elevated TLR4 level in H9c2 cells, and Dioscin partially counteracted this effect of LPS (Figure 3A,B, *p* < .05), but transfection of plasmids overexpressing TLR4 further reversed the inhibitory effect of Dioscin on TLR4 expression in LPS‐induced H9c2 cells (Figure 3A–C, *p* < .01). Moreover, we identified that LPS produced an inhibitory effect on viability of H9c2 cells (Figure 3D, *p* < .001), while Dioscin promoted the viability of LPS‐treated H9c2 cells (Figure 3D, *p* < .01). Of note, overexpressed TLR4 attenuated the promoting effect of Dioscin on LPS‐induced H9c2 cell viability (Figure 3D, *p* < .05). Later, we evaluated the ROS level by DHE staining assay (Figure 3E), and noticed that ROS production was signally enhanced in the LPS‐induced H9c2 cells, which was suppressed by Dioscin. Notably, when LPS‐treated H9c2 cells were transfected with plasmids overexpressing TLR4, the role of Dioscin in ROS production was mitigated (Figure 3E). These results suggested that Dioscin may participate in the oxidative stress of H9c2 cells by regulating TLR4 expression.

### 3.5 Overexpressed TLR4 attenuated the roles of Dioscin in elevating SOD, CAT, and GSH activities and Bcl‐2 level, and diminishing the levels of Bax, MyD88, and p‐p65/p65 in LPS‐treated H9c2 cells

**Figure 4:**
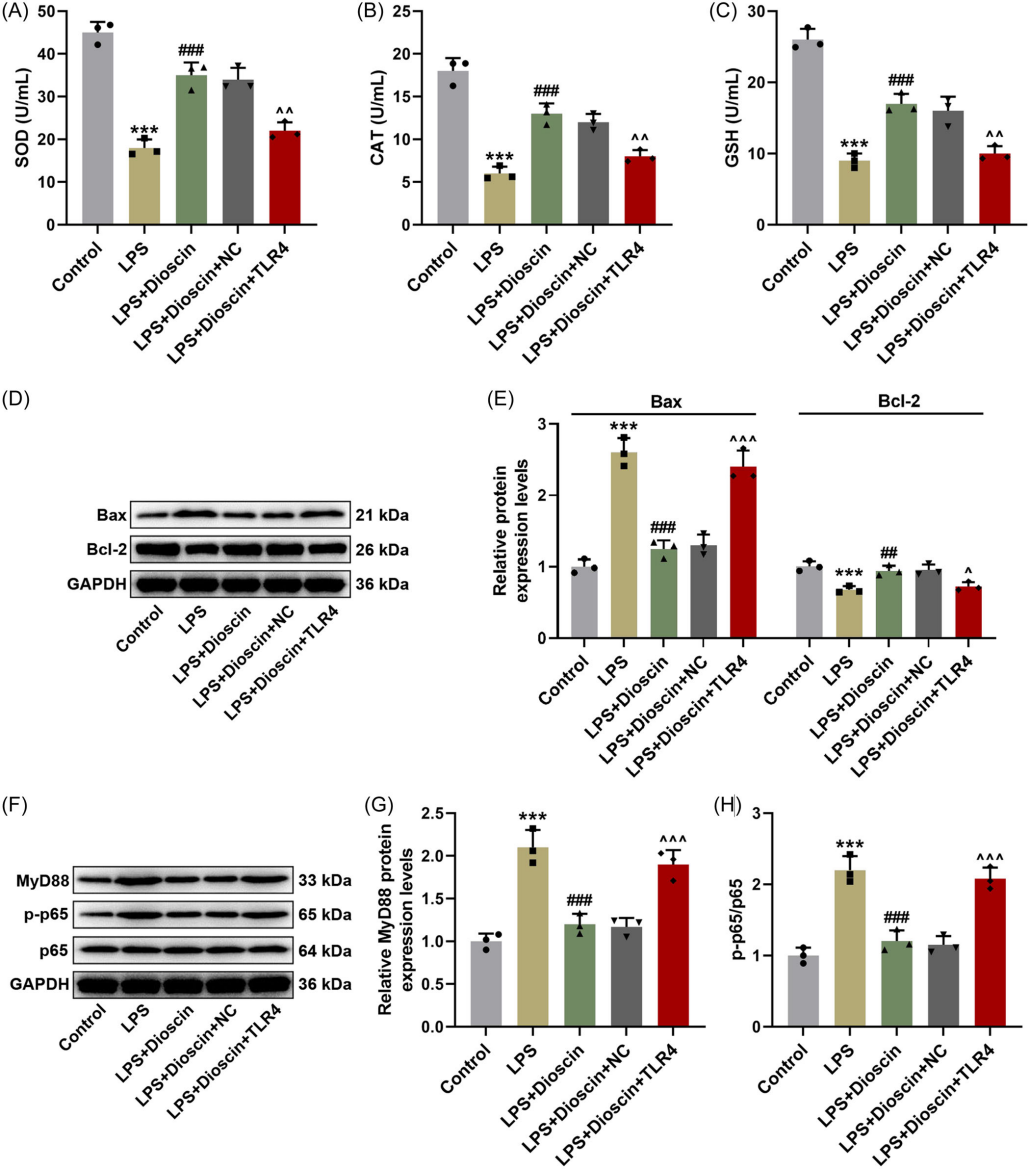
Overexpressed toll‐like receptor 4 (TLR4) attenuated the impacts of Dioscin upon increasing superoxide dismutase (SOD), catalase (CAT), and glutathione (GSH) activities and B‐cell lymphoma‐2 (Bcl‐2) level, and decreasing Bax, MyD88, and phosphorylated‐p65 (p‐p65)/p65 levels in lipopolysaccharide (LPS)‐induced H9c2 cells. (A–C) The activities of SOD (A), CAT (B), and GSH (C) in the treated H9c2 cells were measured using colorimetric assay. (D, E) The expressions of Bcl‐2 and Bax in treated H9c2 cells were detected by Western blot, and glyceraldehyde 3‐phosphate dehydrogenase (GAPDH) was used as an internal control. (F–H) The expressions of MyD88, p‐p65, and p65 in treated H9c2 cells were quantified by Western blot. GAPDH was used as an internal control, and the p‐p65/p65 value was calculated (***p < .001 vs. control; ^##^ *p* < .01; ^###^ *p* < .001 vs. LPS; ^^^ *p* < .05; ^^^^ *p* < .01; ^^^^^ *p* < .001 vs. LPS + Dioscin + negative control [NC]; *n* = 3, one‐way analysis of variance was utilized to compare difference among multiple groups, followed by Tukey post hoc test).

Through in vitro assays, we also proved that LPS obviously inhibited SOD, CAT, and GSH activities in H9c2 cells (Figure 4A–C, *p* < .001), while Dioscin enhanced SOD, CAT, and GSH activities in LPS‐induced H9c2 cells (Figure 4A–C, *p* < .001). However, overexpressed TLR4 partially counteracted this promoting effect of Dioscin on the activities of SOD, CAT, and GSH (Figure 4A–C, *p* < .01). Afterwards, we also examined the expressions of apoptosis‐related factors Bax and Bcl‐2 in LPS‐treated H9c2 cells, and the results of Western blot exhibited that after LPS treatment, the Bax level was notably raised yet Bcl‐2 level was lessened in H9c2 cells (Figure 4D,E, *p* < .001); however, Dioscin reversed the influence of LPS through repressing Bax expression, and boosting Bcl‐2 expression in H9c2 cells (Figure 4D,E, *p* < .01). Besides, transfection with plasmids overexpressing TLR4 was demonstrated to mitigate the effects of Dioscin on Bax and Bcl‐2 levels in LPS‐induced H9c2 cells (Figure 4D,E, *p* < .05). Meanwhile, we found that LPS overtly elevated MyD88 level and p‐p65/p65 value (Figure 4F–H, *p* < .001), whilst Dioscin reversed the promotive effects of LPS on MyD88 expression and p‐p65/p65 value (Figure 4F–H, *p* < .001). Importantly, overexpressed TLR4 weakened the influences of Dioscin via promoting MyD88 expression and p‐p65/p65 value in LPS‐induced H9c2 cells (Figure 4F–H, *p* < .001). These data hinted that Dioscin was implicated in the oxidative stress of LPS‐induced H9c2 cells by mediating TLR4/MyD88/p65 pathway.

## 4 DISCUSSION

Although there is knowledge gap existing in the pathogenesis of SIC, ample evidence has suggested that inflammatory responses, oxidative stress, and cardiomyocyte apoptosis are associated with the development of SIC.^45^, ^46^ Also, Dioscin has been reported to be involved in the protection against LPS‐induced inflammatory kidney injury.^47^ LPS, a major component of the outer membrane of Gram‐negative bacteria, is frequently used to induce SIC in rats as an important immunomodulator.^37^, ^48^ Former research indicated that diseases induced by LPS in rat models are linked to abnormalities in hemodynamic parameters.^49^, ^50^ It has been documented that after treatment with LPS in rats, the HR and HWI are augmented, while LVEF and MAP are lessened,^37^, ^38^ which was consistent with our findings. We also confirmed that Dioscin reversed the effects of LPS on HR, HWI, LVEF, and MAP in SIC rats. In a study regarding sepsis‐induced cardiac dysfunction, LPS has been validated to induce changes in inflammatory responses and myocardial morphology in rats.^51^ In sepsis‐induced myocardial dysfunction in rats, the level of 4‐HNE, a marker of lipid peroxidation, is increased under the induction of LPS.^52^ In the current research, we proved that LPS exacerbated myocardial tissue damage, and increased 4‐HNE level in SIC rat model, the effects of which were reversed by Dioscin, demonstrating the protective effect of Dioscin on rat myocardial tissues.

In animal cardiomyocytes exposed to LPS, some immunosuppressants, such as cyclosporin A, generate inhibitory effects on mPTP opening.^53^, ^54^, ^55^, ^56^ In addition, verbascoside, which has been proved to exert antioxidative and neuroprotective effects, alleviates oxidative stress, inflammatory cell infiltration, and cardiomyocyte apoptosis in LPS‐induced SIC rat model.^57^ In our research, we found that Dioscin attenuated the role of LPS and had a protective effect on rat myocardial tissue, which provides a possibility for the development of drugs targeting immunity and inflammation in SIC diseases based on Dioscin.

Previous evidence revealed the participation of oxidative stress and cardiomyocyte apoptosis in the development of SIC,^45^, ^46^ which was further confirmed in our subsequent assays. Antioxidant enzymes mainly include SOD, CAT, and GSH.^38^ SOD can catalyze the conversion of highly reactive superoxide to hydrogen peroxide, and plays a key role in maintaining the balance of oxidative stress.^58^ CAT makes a profound impact upon protecting cells from the deleterious effects of hydrogen peroxide.^59^ GSH is also one of the most important factors in the cellular antioxidant defense system.^60^ Relevant studies have found that LPS induces myocardial injury by decreasing the activities of SOD, CAT, and GSH.^38^ Liu et al. have demonstrated that Dioscin boosts the activities of SOD, CAT, and GSH in hippocampal neuron damage induced by oxygen–glucose deprivation/reperfusion.^61^ In this work, we revealed that Dioscin reversed the role of LPS in decreasing the activities of SOD, CAT, and GSH in SIC rat model. Besides, Bax and Bcl‐2 are apoptosis‐related factors, of which Bcl‐2 has a significant inhibitory effect on apoptosis, and conversely Bax can promote apoptosis.^62^ Song et al. have indicated that Dioscin inhibits the apoptosis of Doxorubicin‐induced Alpha mouse liver 12 cells.^63^ In this study, we demonstrated for the first time that Dioscin attenuated the influences of LPS through elevating Bcl‐2 level, and dwindling Bax level in SIC rat model. These data manifested that Dioscin generated protective effects by dampening LPS‐induced oxidative stress and cardiomyocyte apoptosis in LPS‐induced SIC rat model.

In addition, it was found that LPS induces an increase in TLR4 protein expression in the lung tissue of mice.^26^ Due to its antioxidant and anti‐inflammatory properties, Dioscin also can inhibit LPS‐induced TLR4 expression.^36^, ^64^ In this study, we identified that LPS promoted TLR4 expression both in vivo and in vitro, and transfection of TLR4 overexpression plasmids reversed the inhibitory effect of Dioscin on TLR4 expression in LPS‐induced H9c2 cells. These findings provide further evidence to support the involvement of Dioscin in antioxidant and anti‐inflammatory mechanisms. In human nucleus pulposus cells, Dioscin attenuates IL‐1β‐induced apoptosis by modulating the TLR4 level.^65^ A large accumulation of ROS has been corroborated to cause oxidative stress damage and excessive apoptosis in cardiomyocytes.^38^ In this experiment, we found through in vitro assays that LPS had an inhibitory effect on viability of H9c2 cells, while Dioscin promoted the viability of LPS‐treated H9c2 cells, and notably overexpressed TLR4 offset the promoting role of Dioscin in the viability. Subsequently, we identified that overexpressed TLR4 weakened the effects of Dioscin on increasing SOD, CAT and GSH activities and Bcl‐2 level, and decreasing Bax level in LPS‐treated H9c2 cells. Combined with the in vivo and in vitro experimental results, it can be found that Dioscin inhibited LPS‐induced oxidative stress and apoptosis in H9c2 cells.

Moreover, a previous study has reported that Dioscin suppresses ischemic stroke‐induced inflammation by TLR4/MyD88/NF‐κB signal pathway in a rat model.^66^ Zeng et al. unveiled that Dioscin prevents LPS‐induced ALI via inhibiting the TLR4/MyD88 signal pathway through upregulation of heat shock protein 70.^35^ In addition, Qi et al. evidenced that Dioscin suppresses TLR4/MyD88 signal pathway to dampen inflammation, oxidative stress and apoptosis.^47^ Interestingly, the regulation of TLR4/MyD88 pathways by Dioscin appears to involve the upregulation of microRNA let‐7i which targets and inhibits TLR4.^47^ In our study, we found that LPS elevated MyD88 level and p‐p65/p65 value, which was reversed by Dioscin; however, overexpressed TLR4 attenuated the effect of Dioscin on diminishing MyD88 expression and p‐p65/p65 value in LPS‐treated H9c2 cells. These data illustrated that Dioscin inhibited the oxidative stress and apoptosis of H9c2 cells by regulating TLR4/MyD88/p65 pathway.

In conclusion, our results authenticated the protective effects of Dioscin against SIC via the TLR4/MyD88/p65 signal pathway, suggesting that Dioscin acts as a new candidate for clinical treatment of SIC.

However, there are several limitations in our study. First, we only evaluated the TLR4/MyD88/p65 signal pathway as the underlying mechanism of Dioscin, but notably, SIC is a complex disease with multifactorial pathogenesis, and there may be other signaling pathways or molecular targets involved in the overall therapeutic effects of Dioscin. Hence, further research is needed to elucidate the mechanisms and potential synergies. Second, based on our animal model, clinical validation for efficacy and safety in humans is warranted. Third, conducting comprehensive toxicity studies and evaluating potential drug interactions are crucial before clinical application of Dioscin.

## AUTHOR CONTRIBUTIONS

**Meng Zhang**: Project administration; writing—original draft. **Deyuan Zhi**: Data curation; formal analysis. **Pei Liu**: Investigation; resources. **Yajun Wang**: Investigation; resources.

## CONFLICT OF INTEREST STATEMENT

The authors declare no conflict of interest.

## ETHICS STATEMENT

This research protocol was approved by the Bioethics Committee of Beijing Friendship Hospital, Capital Medical University (20‐2007). All animal experiments were performed in accordance with the guidelines of the Chinese Animal Care and Use Committee.

## Data Availability

The analyzed datasets generated during the study are available from the corresponding author on reasonable request.
